# Selection of heat stress tolerant wheat genotypes for desert environments

**DOI:** 10.1038/s41598-025-20450-7

**Published:** 2025-10-21

**Authors:** Abdelhalim I. Ghazy, Talal K. Al Ateeq, Eid I. Ibrahim, Hussein Abdel-Haleem, Kotb A. Attia, Omar Azab, Abdullah A. Al-Doss

**Affiliations:** 1 Plant Production Department, College of Food and Agricultural Sciences, King Saud University, 11362 Riyadh, Saudi Arabia; 2 US Arid Land Agricultural Research Center, USDA-Agricultural Research Services, Maricopa, AZ 85138 USA; 3 Department of Biochemistry, College of Science, King Saud University, P.O. Box 2455, 11451 Riyadh, Saudi Arabia

**Keywords:** Heat stress, Molecular markers, Semiarid regions, Wheat, Natural variation in plants, Plant breeding, Plant genetics, Plant physiology, Plant stress responses

## Abstract

High temperature is a critical abiotic stress that severely impacts agricultural productivity, especially in semi-arid and arid regions. This study assesses the phenotypic performance and genetic diversity of twenty advanced wheat genotypes and checks under the field conditions of heat stress for two years. Heat stress led to significant reductions in grain yield and related traits, with an average yield decline of 53.8%. Path analysis revealed a negative impact of heading date on grain yield under stress conditions. Stress indices indicated strong heat tolerance in the genotype YR × Ksu110-240, which showed only a 10.4% reduction in grain yield, whereas DHH3-26 exhibited high sensitivity with a 53.6% reduction. Genetic diversity analysis using 30 Simple Sequence Repeat (SSR) polymorphic markers identified significant marker-trait associations, particularly Xgwm 285 and Xgwm 577, which were strongly linked to heat tolerance related traits. These markers provide valuable tools for marker-assisted selection (MAS), facilitating the breeding of heat-resilient wheat varieties. This study highlights the significance of combining molecular markers with phenotypic assessments to improve wheat adaptation to challenging environmental conditions. The wide genetic diversity offers opportunities for introducing novel alleles into breeding programs, which will be critical for developing wheat varieties that can sustain productivity under increasingly variable and extreme environmental conditions. By using the genetic and phenotypic diversities, breeders can target specific traits and markers to develop heat-resilient wheat varieties, ensuring food security in regions threatened by rising global temperatures.

**Supplementary Information:**

The online version contains supplementary material available at 10.1038/s41598-025-20450-7.

## Introduction

Wheat (*Triticum aestivum* L.) is one of the most vital food crops globally. Despite its critical role in global food security, wheat production is increasingly affected by high heat stress, an abiotic stress factor that is exacerbated by the ongoing acceleration of hot and dry weather. Rising temperatures, particularly during sensitive growth stages such as flowering and grain filling, have been shown to negatively affect wheat productivity by reducing grain size, grain number, and overall yield^1,2^. This reduction is particularly severe in wheat-growing regions where late sowing exposes crops to high temperatures during the reproductive phase, leading to significant decreases in both grain and biological yields^3^. As climate uncertainty intensifies, the development of heat-tolerant wheat varieties has become a top priority for breeders and farmers. High heat tolerance is a complex trait and could be assayed by secondary genetic and physiological traits such as grain yield, spike number, days to heading, and biological yield^4,5^. In heat-stressed environments, the phenotypic variability among genotypes becomes more pronounced, allowing for the identification of genotypes that possess inherent heat tolerance. This is a crucial step toward ensuring wheat sustainability in regions highly vulnerable to climate uncertainty^6^. One of the most effective approaches for identifying heat-tolerant genotypes is identifying molecular markers associated with traits related to heat stress. Among the various molecular markers, Simple Sequence Repeats (SSRs) have proven to be particularly valuable to detect extensive genetic variation within and between wheat genotypes^7^. The SSR markers have been widely employed in wheat breeding programs to identify genotypes with desirable traits such as heat tolerance^8^. SSR markers were employed to assess genetic variability and validate quantitative trait loci (QTLs) associated with heat tolerance, which will aid in the development of more robust breeding strategies to mitigate the impacts of heat stress on wheat productivity^9^. This approach enables the classification of wheat genotypes based on their phenotypic performance under various environmental conditions, including heat stress^10^. The use of SSR markers enables breeders to efficiently select tolerant genotypes thereby accelerating the development of heat-tolerant wheat cultivars^11^ with favorable agronomic traits when grew under heat-stressed conditions. The aim of the current study was to evaluate the genetic diversity of heat-tolerant wheat genotypes and assess the stability of wheat genotypes under heat stress conditions. The specific objectives were to (1) analyze the impact of heat stress on important agronomic traits such grain and biological yields and yield components, (2) assess the variability in heat tolerance among different wheat genotypes, (3) identify potential correlations between the stress indices and agronomic traits to determine which traits contribute to heat tolerance, and (4) Identify heat tolerant genotypes and molecular markers for further breeding cycle of improving wheat productivity in semi-arid environment.

## Results

### Phenotypic analysis of heat tolerance in wheat genotypes

Late planting date of wheat genotypes exposed them to high heat stress at heading and grain filling stages, which can hinder grain filling and reduce overall yield. Ensuring timely planting is essential to prevent negative impacts on crop growth and yield. Variance analysis showed significant effects of year (Y), planting date (PD), and genotype (G) on the measured traits (Table 1). Grain yield (GY) was significantly influenced by Y, PD, and G, along with their interactions. Similarly, biological yield (BY) was significantly affected by G, PD, and Y, with a significant interaction observed only between G and Y. In contrast, the number of spikes (NS) was significantly influenced by G, while the interaction between PD and Y was non-significant (Table 1).

**Table 1:** Mean squares of analysis of variance of spike length (SL), number of Kernels (GS), 1000 kernel weigh (KW), plant height (PH), Days to Heading (DHE), Number of Spikes per 0.25 m^2^ (NS), Biological Yield (BY), and Grain Yield (GY) for 20 whet genotypes planted under non heat stress and heat stress conditions.

SOV	SL	GS	KW	PH	DHE	NS	BY	GY
Year(Y)	62.53*	1467.68*	1113.27*	57.62^ ns^	1876.00*	3.75^ ns^	7,161,330*	2,560,060*
Planting date (D)	11.14*	265.23^ ns^	3547.40**	1307.60**	3896.20**	40.02^ ns^	88,435,200**	27,406,890**
Genotype(G)	27.14**	422.76**	90.59**	961.66**	157.48**	179.14**	4,398,620**	629,260**
Y*D	2.97^ ns^	1021.35*	4.68^ ns^	34.05^ ns^	127.60**	400.42*	3627030^ ns^	226870^ ns^
Y*G	1.87^ ns^	82.15^ ns^	24.92^ ns^	29.68*	22.81**	84.81^ ns^	1,163,060**	140400^ ns^
D*G	1.39^ ns^	44.84^ ns^	22.93^ ns^	31.21*	42.87**	97.41*	750310^ ns^	160370^ ns^
Y*D*G	3.89**	120.02**	15.99^ ns^	40.53**	9.22**	37.88^ ns^	933290^ ns^	185,920*

*, ** significant at 0.05 and 0.01 levels, respectively. ^ns^non-significant.

Overall, growing wheat genotypes under heat stress had the largest impact on grain yield and yield components. This highlights the importance of breeding for heat tolerance to mitigate yield losses in wheat production. In the present study, biological and grain yields decreased from 17,099.2 kg ha^−1^ and 6217.9 kg ha^−1^, respectively under normal non-stress condition to 11,760.7 kg ha^−1^ and 3681.3 kg ha^−1^ under stress condition (Table 2). For example, Klassic × Ksu105-11 produced 6527 kg ha^−1^ grain when planted under no heat stress conditions, while under high heat stress conditions produced 4382 kg ha^−1^. The same genotype produced less biological yield under high heat stress (13,425 kg ha^−1^) compared to non-stress conditions (18,051 kg ha^−1^). The impact of high temperature was observed to reduce the number of days to heading for the heat-tolerant KSU genotypes (Klassic × ksu 105-11 and Klassic × Ksu105-213). The Klassic × Ksu105-213 number of days to heading decreased from 75.3 to 71.3 days (Table 2). For the number of spikes in Klassic × Ksu105-213 plant growing under high heat stress conditions and no-stress conditions were 33.5 and 27.7 spikes per 0.25 m^2^, respectively. Growing conditions affected the number of grains per spike as well, where the number of grains per spike of Klassic × Ksu105-213 dropped from 53.7 to 37.7 grains when grown under no-stress and high heat stress conditions, respectively. That reflected on the drop in 1000 grain weight from 47.2 to 37 g respectively (Table 2).

**Table 2:** Means of grain yield and yield components of 20 wheat genotypes growing no-stress (NS) and high heat stress (HT) conditions.

	SL		GS		KW		PH		DHE		NS		BY		GY	
	NS	HT	NS	HT	NS	HT	NS	HT	NS	HT	NS	HT	NS	HT	NS	HT
Lang × Ksu105-87	8.7	8.5	37.1	46.3	42	41.6	92.4	90	70.6	59	34.6	34.3	18,180	13,350	7288	4126
Lang × Ksu105-47	8.8	8.6	36.9	39.4	40.4	35.1	94.4	88.6	70.2	65	34	39.3	19,785	12,300	6124	4139
YR × Ksu110-190	11.9	9.8	43.3	43.5	46	36.9	75.9	73.2	69.2	63.5	29.7	32.8	14,311	9165	5285	2571
YR × Ksu110-240	11.3	10.2	38.6	39	46	39.7	77.9	80.8	69.3	64.3	32.8	34.2	12,833	10,725	4969	3368
YR × Ksu110-277	12.5	10.4	42.3	35.6	45.4	38.5	82.3	77.6	75.3	65.8	30	33.5	15,863	10,425	5644	3171
YR × Lang-4	8.4	9	34.1	42.9	41.3	32.8	95.8	90.9	78.7	69.5	35	26.5	17,827	12,450	6250	4286
YR × Lang-15	9.5	9.1	33.2	39.3	47.2	38.3	95.2	88.6	79.7	70	33	31.7	17,681	12,600	7376	4527
YR × Lang-30	9	8.4	39.7	40.4	44.1	34.7	93.9	88.9	77.5	69.8	32.5	36	15,476	12,675	6427	3858
YR × Lang-60	9	8.6	39.8	40.5	45.9	35.2	91.9	88	77.5	69.7	34	31.8	19,598	12,075	7300	4384
YR × Lang-66	9.7	8.8	41.4	42.5	43.7	32.9	97.1	93.4	77	69.7	39.7	37.5	18,241	12,675	7280	3778
Klassic × Ksu105-11	11.3	10.7	46.4	46.1	44.5	37.4	94.3	94.2	76.7	72.8	29.2	28.2	18,051	13,425	6527	4382
Klassic × Ksu105-213	10.5	11.3	37.7	53.7	47.2	37	94.9	90.7	75.3	71.3	33.5	27.7	18,356	13,800	6743	4501
Lang	9.4	9.1	47.7	46.3	40.4	32.2	94	91	79.7	74.5	30.2	40.5	15,947	12,900	5803	4304
YecoraRojo	10.9	9.9	38.6	41.4	45.4	40.3	75.9	69.8	71	66.3	31.8	36.2	15,564	10,200	5774	3793
Klassic	10.1	9.2	38.9	41.7	42.6	40	76.8	71	73.2	66.8	31	38.3	13,417	9075	4990	2832
Ksu105	10.5	11	42.4	47.5	45.3	35.3	97.7	90.5	75.8	71.8	39.3	34	19,851	13,350	7312	4098
Ksu110	12.8	12.1	57.3	58.4	44	42	76.1	73.2	72.2	67.8	31.5	22.3	15,647	9525	5743	3125
Ksu115	14.7	14.5	58.6	59.2	45.7	41.8	85.2	80.2	77.8	71.3	24.8	23	16,309	9225	5498	2909
DHH3-26	11.6	11	42.6	46.7	36.6	30	108.8	96.7	74	84.3	38	38.3	21,699	13,725	6307	1850
DHH4-76	10	9.7	41.6	41.3	41.9	33.5	101.4	91	77	74.3	29.8	41.2	17,350	11,550	5719	3633
LSD_0.05_	1		6		3.4		3.3		1.4		5.9		1927.4		847.9	

SL: spike length; GS number of kernel per spike; KW: 1000 kernel weight (g); PH: plant height (cm); DHE: number of days to heading; NS: number of spike per ¼ square meter; BY: biological yield weight (Kg Ha^−1)^; GY grain yield (Kg Ha^−1^).

**Fig. 1:**
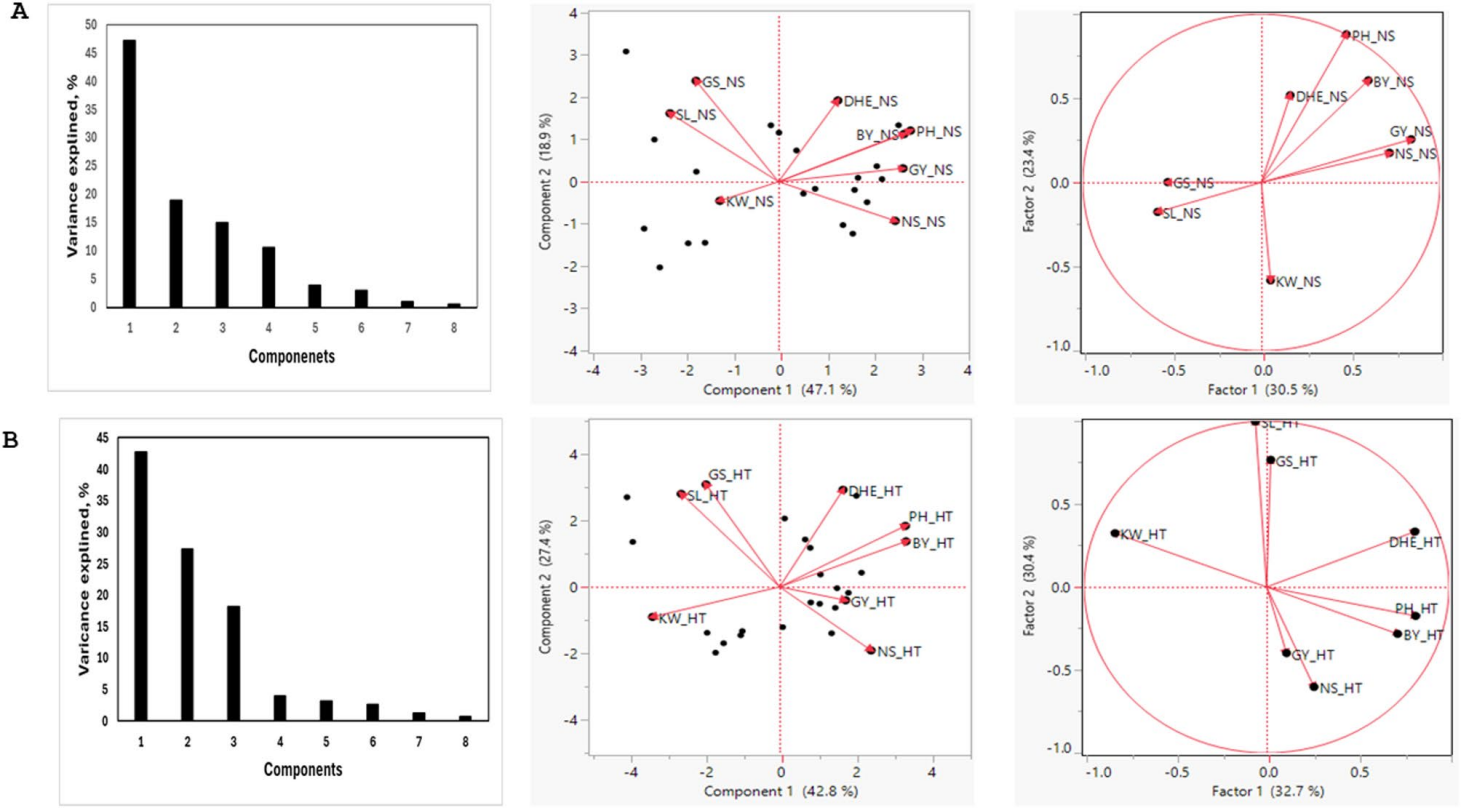
Principle Components Analysis for yield and yield components ((spike length (SL), number of Kernels (GS), 1000 kernel weigh (KW), plant height (PH), Days to Heading (DHE), Number of Spikes per 0.25 m^2^ (NS), Biological Yield (BY), and Grain Yield (GY)) for 20 wheat genotypes grown under no-stress conditions (NS, Fig. 1A) and high heat stress conditions (HT, Fig. 1B)**.**

The correlation analyses (Table 3) illustrated the relationships among grain yield and its components traits for wheat genotypes grown under both non-stress and heat-stress conditions. Under non-stress conditions, biological yield showed a strong positive correlation with grain yield, indicating that higher biomass directly contributes to increased grain production under favorable growing conditions. Similarly, 1000 grains weight and spike length positively correlated grain yield, suggesting that both traits are enhancing yield potential in the absence of abiotic stress. Under high heat stress, the significant correlations between 1000 grains weight and spike length and grain yield were weakened or altered (Table 3), indicating that heat stress affects the effectiveness of 1000 grains weight and spike length traits to enhance grain yield. The accumulation of biomass and kernel weight is more adversely affected by high temperatures, leading to reduced grain yield. Interestingly, days to heading negatively correlated with grain, even not significant, under high heat stress, while positively corrected under non-stress conditions, indicating that early-flowering genotypes could be an important factor to maintain higher yields under stressful environments. That emphasizes the importance of early reproductive development as a strategy to escape severe effects of high heat during flowering and grain filling stages. The principal component analysis (PCA) explained eight principal components explained the total variations in genotypes grown under high heat stress and non-stress conditions (Fig. 1). The significant first components where PC1 explained 47.1% of the total variations, and PC2 accounted for 18.9% of the total variance for yield and yield components traits estimated for genotypes grown under non stress conditions (Fig. 1A), while PC1 and PC2 explained 42.8% and 27.4% respectively for the same traits under high heat stress conditions (Fig. 1B). Based on the dissimilarity and homogeneity among traits, PCA biplot and factor analyses clustered yield and yield components traits into four clusters (Fig. 1) where kernel weight (KW) assembled a cluster; number of kernel per spike (GS) and spike length (SL) clustered together, grain yield (GY) and number of spikes (NS) correlated together and plant height ( PH), heading date (DHE) and biological yield (BY) correlated in one cluster.

**Table 3:** Pearson correlation coefficients among grain yield and yield components of 20 wheat genotypes grown under stress (upper) and no stress (lower) conditions.

		Stress conditions							
Non-stress conditions		SL	GS	KW	PH	DHE	NS	BY	GY
	SL		**0.4**	**0.06**	**− 0.14**	**0.42**	**− 0.36**	**− 0.14**	**− 0.29**
			*0.01*	*0.71*	*0.39*	*0.01*	*0.03*	*0.4*	*0.08*
	GS	**0.73**		**0.45**	**− 0.09**	**− 0.26**	**− 0.52**	**− 0.07**	**0.1**
		< *.0001*		*0*	*0.57*	*0.11*	*0*	*0.69*	*0.56*
	KW	**0.03**	**0.08**		**− 0.57**	**− 0.69**	**− 0.44**	**− 0.42**	**0.09**
		*0.85*	*0.65*		*0*	< *.0001*	*0*	*0.01*	*0.57*
	PH	**− 0.31**	**− 0.12**	**− 0.33**		**0.45**	**0.13**	**0.77**	**0.27**
		*0.06*	*0.49*	*0.04*		*0*	*0.44*	< *.0001*	*0.1*
	DHE	**0.13**	**0.13**	**− 0.38**	**0.31**		**0.22**	**0.31**	**− 0.27**
		*0.44*	*0.45*	*0.02*	*0.06*		*0.17*	*0.06*	*0.1*
	NS	**− 0.41**	**− 0.32**	**0**	**0.33**	**− 0.25**		**0.27**	**0.01**
		*0.01*	*0.05*	*0.98*	*0.04*	*0.13*		*0.1*	*0.97*
	BY	**0**	**− 0.13**	**− 0.4**	**0.48**	**0.54**	**0.03**		**0.51**
		*0.98*	*0.45*	*0.01*	*0*	*0*	*0.87*		*0*
	GY	**− 0.33**	**− 0.24**	**0.12**	**0.43**	**0.32**	**0.24**	**0.69**	
		*0.04*	*0.14*	*0.45*	*0.01*	*0.05*	*0.15*	< *.0001*	

Upper bold values indicate correlation coefficients; lower values represent corresponding *p* values.

**Fig. 2:**
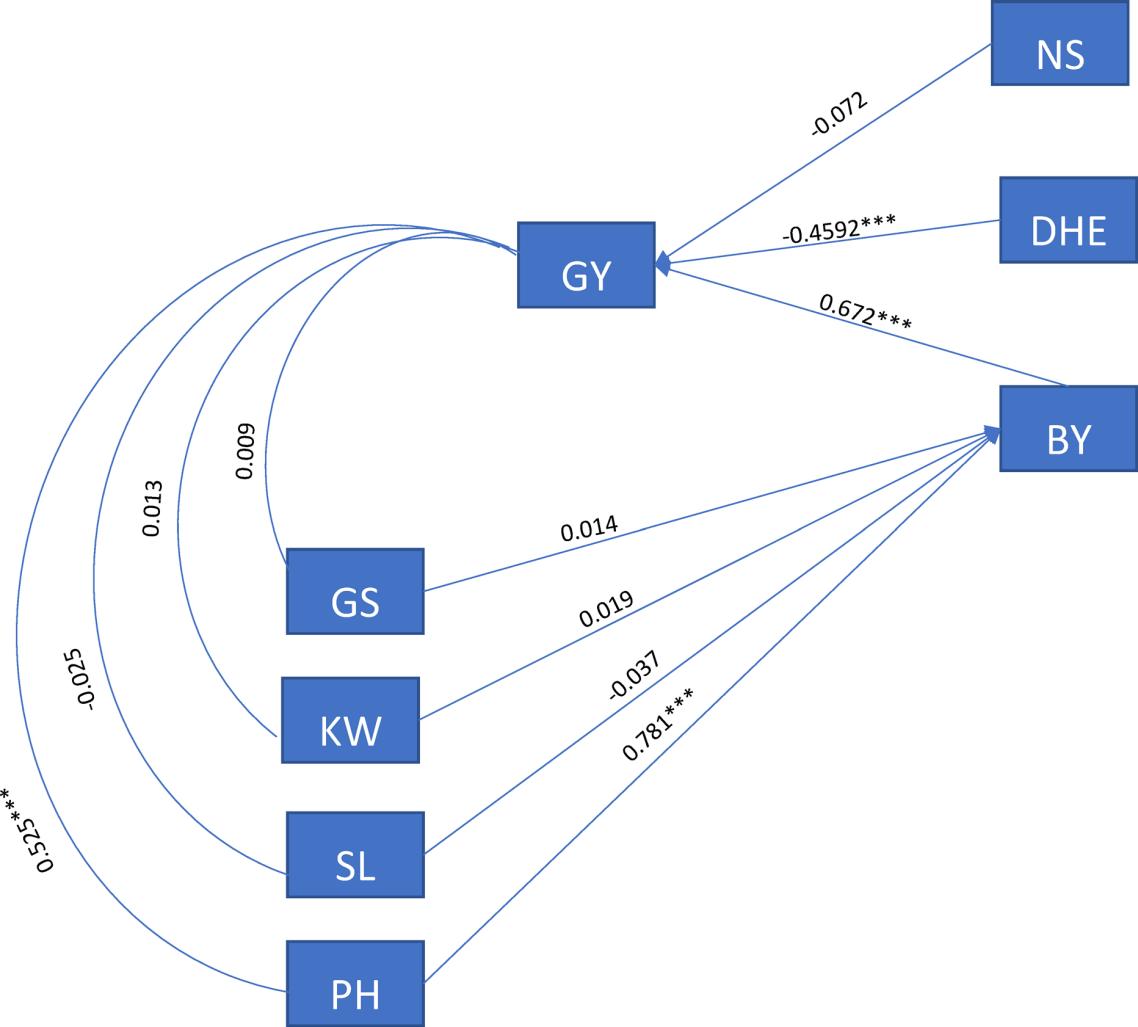
Path analysis for yield and yield components (spike length (SL), number of Kernels (GS), 1000 kernel weigh (KW), plant height (PH), Days to Heading (DHE), Number of Spikes per 0.25 m^2^ (NS), Biological Yield (BY), and Grain Yield (GY)) for 20 wheat genotypes grown under high heat stress conditions**.**

Path analysis was conducted to partition the correlation coefficients into direct and indirect effects among grain yield and its related traits for the wheat genotypes grown under stress conditions (Fig. 2). Results showed that plant height has a positive direct effect (0.781, *p* < 0.0001) on the biological yield which reflected on positive indirect effect (0.525, *p* < 0.0001) of the grain yield. Even if it is not significant, spike length had negative direct effect on biological yield thus negative indirect effect on grain yield. Heading date as well found to have a negative direct effect (− 0459, *p* = 0.0001) on grain yield. In agreement with correlation analysis, path analyses indicated that exposing wheat plants to high heat during the heading and grain filling stages could affect grain yield through affecting yield component traits such as spike length.

To understand the responses of the studied wheat genotypes to high heat stress, nine stress indices were estimated to predict the better performance genotype in terms of lower yield loss, greater stability across environments, and resilience in key agronomic traits under heat stress. The nine stress indices including harmonic mean of yield (HM), geometric mean productivity (GMP), yield reduction (YR), stress tolerance (TOL), yield index (YI), yield stability index (YSI), stress Tolerance index (STI), stress tolerance efficiency (STE) and relative stress index (RSI) were used in the current study. Those indices were used to predict the strong candidates for future breeding programs are aiming at enhancing the high heat tolerance in wheat. Correlation analysis showed strong positive correlations between grain yield under stress and HM, GMP, YI, YSI, STI, STE and RSI. There were strong negative correlations between yield under high heat stress and both of YR and TOL (Table 4).

**Table 4:** Correlation coefficients of stress indices for grain yield (Kg Ha^−1^) for no stress (GY_NS) and high heat stress (GY_HT) and stress indexes for 20 wheat genotypes grown under stress and no-stress conditions.

	GY_HT	HM	GMP	YR	TOL	YI	YSI	STI	STE	RSI
GY_NS	**0.589**	**0.733**	**0.814**	**0.052**	**0.519**	**0.589**	**− 0.052**	**0.837**	**− 0.052**	**− 0.052**
	*0.006*	*0*	< *.0001*	*0.827*	*0.019*	*0.006*	*0.827*	< *.0001*	*0.827*	*0.826*
GY_HT		**0.978**	**0.947**	**− 0.774**	**− 0.385**	**1**	**0.774**	**0.933**	**0.774**	**0.774**
		< *.0001*	< *.0001*	< *.0001*	*0.094*	< *.0001*	< *.0001*	< *.0001*	< *.0001*	< *.0001*
HM			**0.992**	**− 0.634**	**− 0.198**	**0.978**	**0.634**	**0.984**	**0.634**	**0.634**
			< *.0001*	*0.003*	*0.403*	< *.0001*	*0.003*	< *.0001*	*0.003*	*0.003*
GMP				**− 0.533**	**− 0.073**	**0.948**	**0.533**	**0.998**	**0.533**	**0.533**
				*0.016*	*0.76*	< *.0001*	*0.016*	< *.0001*	*0.016*	*0.016*
YR					**0.878**	**− 0.774**	**− 1**	**− 0.495**	**− 1**	**− 1**
					< *.0001*	< *.0001*	< *.0001*	*0.026*	< *.0001*	< *.0001*
TOL						**− 0.385**	**− 0.878**	**− 0.03**	**− 0.878**	**− 0.878**
						*0.094*	< *.0001*	*0.898*	< *.0001*	< *.0001*
YI							**0.774**	**0.933**	**0.774**	**0.774**
							< *.0001*	< *.0001*	< *.0001*	< *.0001*
YSI								**0.495**	**1**	**1**
								*0.026*	< *.0001*	< *.0001*
STI									**0.495**	**0.495**
									*0.026*	*0.026*

HM: Harmonic Mean of yield; GMP; Geometric Mean Productivity; YR: Yield Reduction; TOL: Stress tolerance; YI: Yield index; YSI: Yield Stability index; STI: Stress Tolerance index; STE: Stress Tolerance Efficiency and RSI: Relative Stress index. Significant values are in [bold/italics].

**Fig. 3:**
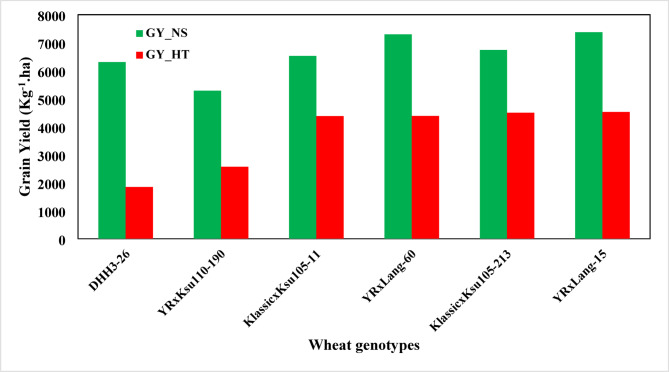
Yield reduction in high yielding group (Klassic × Ksu105-11, YRxLang-60, and Klassic × Ksu105-213 and YR × Lang-15) and low yielding group (DHH3-26 and YR × Ksu110-190) when grown under stressed (GY_HT) and non-stressed (GY_NS) conditions.

Taking in account the stress indices values and yield performance, the new advanced germplasm could be classified as low and high groups. For example, Klassic × Ksu105-11, YRxLang-60, and Klassic × Ksu105-213 and YR × Lang-15, were the highest genotypes group in grain yield under high heat stress, while DHH3-26 and YR × Ksu110-190 were the lowest group (Table 5, Fig. 3).

**Table 5:** Means of grain yield of 20 wheat genotypes grown under stress and no-stress conditions and their stress indices.

Genotype	GY_NS	GY_HT	HM	GMP	YR	TOL	YI	YSI	STI	STE	RSI
DHH3-26	6307	1850	2861	3416	70.672	4458	0.503	0.293	0.302	29.328	0.495
YR × Ksu110-190	5285	2571	3459	3686	51.36	2715	0.698	0.486	0.352	48.64	0.822
Klassic	4990	2832	3613	3759	43.25	2158	0.769	0.568	0.366	56.75	0.958
Ksu115	5498	2909	3805	3999	47.095	2589	0.79	0.529	0.414	52.905	0.894
Ksu110	5743	3125	4048	4237	45.581	2618	0.849	0.544	0.464	54.419	0.919
YR × Ksu110-277	5644	3171	4060	4230	43.824	2473	0.861	0.562	0.463	56.176	0.949
YR × Ksu110-240	4969	3368	4015	4091	32.209	1600	0.915	0.678	0.433	67.791	1.145
DHH4-76	5719	3633	4443	4558	36.468	2085	0.987	0.635	0.538	63.532	1.073
YR × Lang-66	7280	3778	4974	5244	48.105	3502	1.026	0.519	0.712	51.895	0.876
YecoraRojo	5774	3793	4578	4680	34.317	1982	1.03	0.657	0.567	65.683	1.109
YR × Lang-30	6427	3858	4821	4979	39.975	2569	1.048	0.6	0.641	60.025	1.014
Ksu105	7312	4098	5252	5474	43.959	3214	1.113	0.56	0.775	56.041	0.947
Lang × Ksu105-87	7288	4126	5269	5484	43.384	3162	1.121	0.566	0.778	56.616	0.956
Lang × Ksu105-47	6124	4139	4940	5035	32.419	1985	1.124	0.676	0.656	67.581	1.141
YR × Lang-4	6250	4286	5085	5176	31.424	1964	1.164	0.686	0.693	68.576	1.158
Lang	5803	4304	4942	4998	25.839	1500	1.169	0.742	0.646	74.161	1.253
KlassicxKsu105-11	6527	4382	5243	5348	32.871	2146	1.19	0.671	0.74	67.129	1.134
YR × Lang-60	7300	4384	5478	5657	39.95	2916	1.191	0.601	0.828	60.05	1.014
Klassic × Ksu105-213	6743	4501	5398	5509	33.246	2242	1.223	0.668	0.785	66.754	1.127
YR × Lang-15	7376	4527	5611	5778	38.627	2849	1.23	0.614	0.864	61.373	1.037

GY_NS and GY_HT: grain yield (kg Ha^−1^) for no stress and high heat stress respectively. HM: Harmonic Mean of yield; GMP; Geometric Mean Productivity; YR: Yield Reduction; TOL: Stress tolerance; YI: Yield index; YSI: Yield Stability index; STI: Stress Tolerance index; STE: Stress Tolerance Efficiency and RSI: Relative Stress index.

The yield reduction (YR index) in the high yielding group averaged 36%, while the YR in the lowest yielding group reached 61%. The genotype, DHH3-26, exhibited the highest in YR value (70.6%) with low HM, YI, YSI, STI, STE and RSI values and high value of TOL indicated that genotype is affected by the increase in air temperature during heading and grain filling stages. In contrast KlassicxKsu105-11 and Klassic × Ksu105-213 showed lower yield reduction (32.8 and 33,3%, respectively) with high HM, YI, YSI, STI, STE and RSI values and low values of TOL. Another genotype, YR × Ksu110-240, exhibited superior performance under stress conditions, that maintained relatively high biological and grain yields, minimal reductions in kernel weight and early heading date (Table 3). This genotype showed high HM, YI, YSI, STI, STE and RSI values (Table 5). The high values of stress indices and stability indicated the high tolerance of those genotypes to high heat stress, and the suitability to be used as parental material in the future breeding programs.

**Fig. 4:**
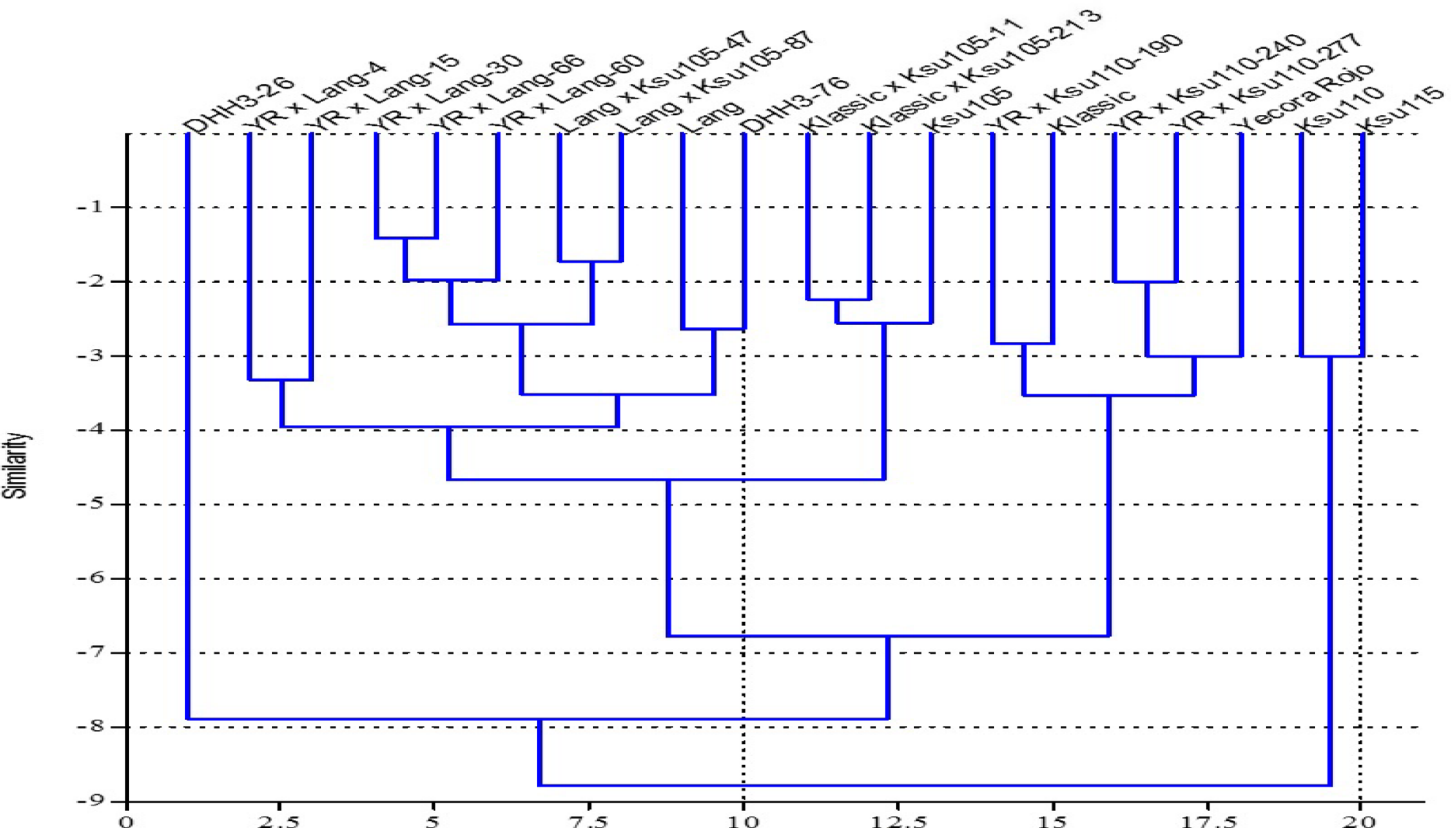
Jaccard similarity coefficient of 20 wheat genotypes, generated by ten agronomic traits.

Jaccard similarity coefficient was used to measure the similarity among the 20 wheat genotypes grown under heat stress conditions genotypes based on their performance in key traits such as grain yield, number of spikes, and days to heading (Fig. 4). Analysis clustered the tested wheat genotypes into three clusters. The first cluster included DHH3-26, which was isolated from the other groups, indicating that it may possess unique traits valuable for introducing genetic diversity into breeding programs. The second cluster consisted of two genotypes, KSU-110 and KSU-115, which formed smaller group. The classification of KSU-110 and KSU-115 in the same cluster, combined with their poor phenotypic performance, indicates their vulnerability for heat stress growing conditions. However, their genetic distinctiveness could be valuable for introducing novel diversity into breeding lines. The third cluster, the largest, contained three subgroups. The first subgroup included Yecora Rojo, Yecora Rojo × KSU-110-277, Yecora Rojo × KSU-110-240, Klassic, and Yecora Rojo × KSU-110-190. Genotypes in this subgroup demonstrated consistent reductions in agronomic traits like days to heading, spike number, and biological yield. Phenotypic similarity across these genotypes supports their close clustering in the dendrogram, as they respond similarly to heat stress, showing moderate heat tolerance. The second subgroup comprised KSU105, Klassic × KSU-105-213, and Klassic × KSU-105-11 genotypes. The third subgroup consisted of the remaining genotypes. This subgroup exhibited stable performance across seasons for traits like biological yield. These genotypes’ moderate reductions in yield and other traits under heat stress reflect their potential to serve as parental lines for breeding programs focused on enhancing heat tolerance.

### Genetic diversity and marker-trait associations analyses

**Fig. 5:**
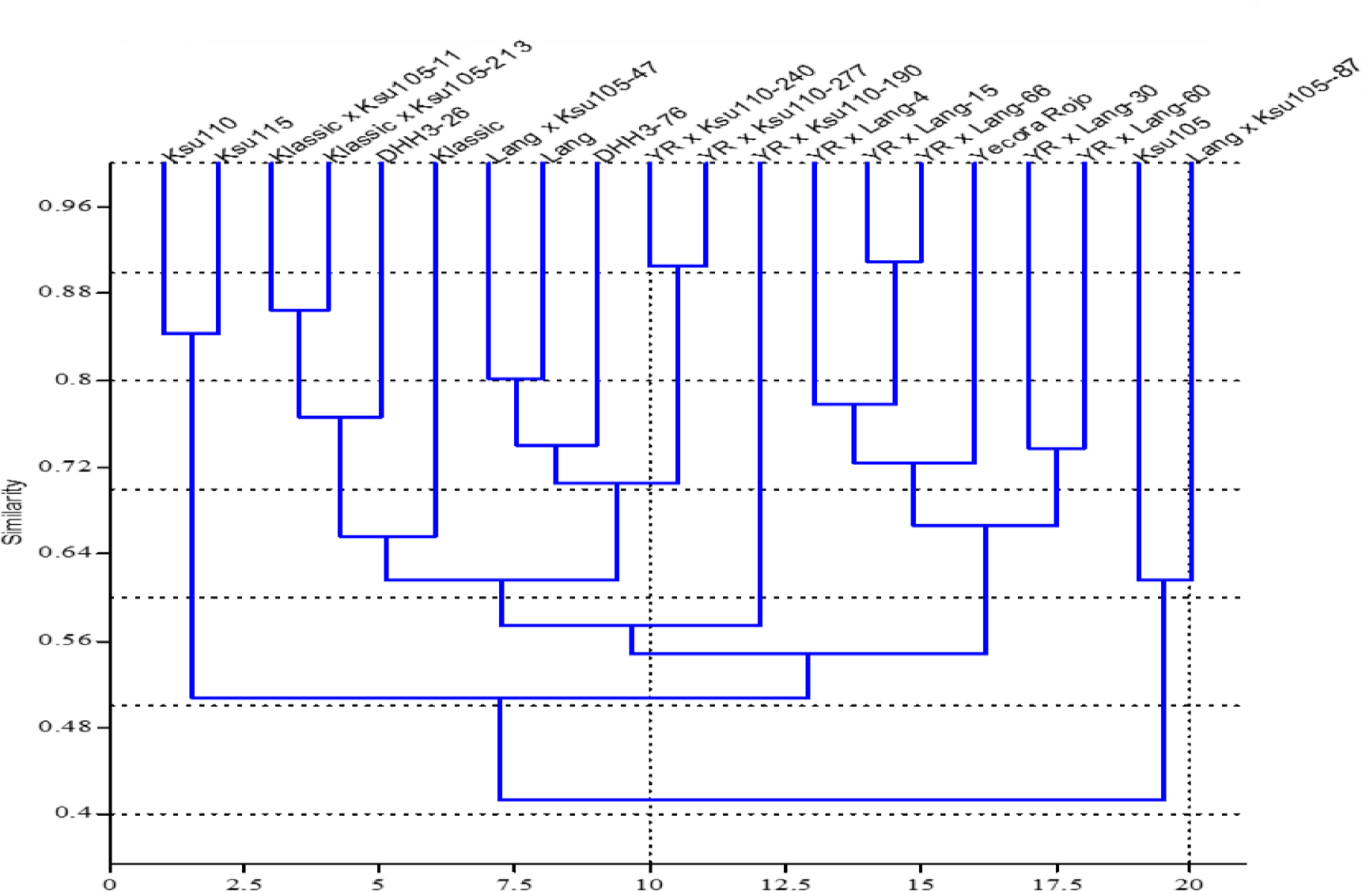
Jaccard similarity coefficient of 20 wheat genotypes, generated by 30 polymorphic SSR markers.

In this study, genetic analysis of terminal heat tolerance was conducted to validate QTLs associated with stress-related traits in wheat. A total of 30 Simple Sequence Repeat (SSR) markers distributed across multiple chromosomes were successfully used to assess genetic diversity among 20 wheat genotypes, all of which amplified polymorphic bands, indicating clear genetic differentiation among the genotypes.Furthermore, 13 SSR markers out of these 30 SSR primers were validated as linked to heat tolerance (Table 6). The broad range of genetic variation depicted in the dendrogram underscores the capability of the 30 SSR markers to reflect the genetic differences among these genotypes (Fig. 5). Genotypes located on longer branches are genetically more distinct, which is crucial for the introduction of new alleles associated with important traits such as stress tolerance and yield stability in breeding programs. Jaccard similarity coefficient values range from 0.18 to 1.00, indicating differing levels of genetic diversity among the genotypes. The analysis identified three distinct clusters among the 20 wheat genotypes. The first cluster, the smallest, consists of two genotypes (KSU-110 and KSU-115). The second cluster contains the majority of genotypes and is further divided into four subclusters, encompassing a total of sixteen genotypes. This clustering suggests a shared genetic background, likely reflecting common parentage or selection history. The third cluster includes two genotypes (KSU-105 and Lang × KSU-105-87) (Fig. 4).

**Table 6:** The proportion of phenotypic variation explained by molecular markers (R2) for the significant associations between SSR markers and spike length (SL), number of kernel per spike (GS), 1000 kernel weight (KW g), plant height (PH, cm), number of days to heading (DHE), number of spike per ¼ square meter (NS) and biological (BY) and grain (GY) yields (kg ha^−1^). *, **, *** significancy level at *P* = 0.5, 0.01 and 0.001 respectively.

Chromosome	Marker	SL	GPS	GW	PH	DHE	NS	BY	GY
1B	Xbarc 128-1B-210 bp	0.027	0.002	0.26*	0.03	0.27*	0.12	0.004	0.05
1B	Xbarc 128-1B-240 bp	0.03	0.003	0.22*	0.0003	0.12	0.1	0.013	0.15
1B	Xgwm 11-1B-280 bp	0.2*	0.1	0.02	0.042	0.01	0.005	0.11	0.23*
1B	Xgwm 11-1B-400 bp	0.03	0.1	0.0001	0.001	0.06	0.027	0.021	0.044
3B	Xgwm 285-3B-400 bp	0.38**	0.44**	0.08	0.056	0.05	0.22*	0.08	0.27*
5A	Xbarc 142-5A-300 bp	0.02	0.04	0.003	0.007	0.09*	0.028	0.0004	0.0004
6B	Xgwm 577-6B-160 bp	0.1*	0.18	0.41**	0.71***	0.1	0.1	0.7***	0.26*
6B	Xgwm 577-6B-180 bp	0.1*	0.18	0.41**	0.71***	0.1	0.1	0.7***	0.26*
7B	Xgwm 611-7B-170 bp	0.06	0.017	0.15	0.06	0.02	0.26*	0.01	0.04
7B	Xgwm 611-7B-190 bp	0.21*	0.12	0.005	0.1	0.07	0.03	0.064	0.00001
7D	Xgwm 617-7D-130 bp	0.1	0.24*	0.002	0.06	0.17	0.00005	0.041	0.0002

To valid QTLs for stress-related traits in wheat and use those markers in our breeding program, genetic analysis of terminal heat tolerance was conducted using seventeen SSR markers, which were dispersed throughout the six chromosomes and were associated with heat stress tolerance in wheat^12,13^. The polymorphism information content (PIC) values calculated for the SSR markers ranged from 0 to 0.38, with Xgwm 577 and Xwmc 170 showing the highest PIC values at 0.38, indicating strong allelic diversity and high informativeness for these markers.

The single marker analysis between SSR markers and key agronomic traits revealed significant marker-trait associations, indicating the potential use of these markers in marker-assisted selection (MAS) for high heat tolerance in wheat breeding programs (Table 6) and the marker classes means of significant markers classes associated with high heat tolerance (Supplementary table 5). Notably, marker Xbarc 128-1B (210 bp) exhibited a significant association with 1000-kernels weight (R^2^ = 0.26, *p* = *0.02*) and days to Heading (R^2^ = 0.27, *p* = *0.017*), suggesting its relevance in influencing these critical yield-related traits. Another allele of the same marker, Xbarc 128-1B (240 bp), was also linked to 1000-kernel weight (R^2^ = 0.22, *P* = 0.034), further supporting its importance for breeding programs focused on improving grain yield and early maturity. Marker, Xgwm 577-6B (160 bp), was associated with multiple agronomic traits, including 1000-kernel weight (R^2^ = 0.41, *p* = *0.002*), plant height (R^2^ = 0.71, *p* = 0.000003), and biological yield (R^2^ = 0.70, *p* = 0.000003). These strong associations suggested that Xgwm 577-6B (160 bp) may play a pivotal role in controlling genes that regulate grain yield and yield components, making it a valuable marker for identifying high-yielding wheat genotypes. In addition, marker Xgwm 611-7B (170 bp) showed a significant correlation with spike number in area (R^2^ = 0.26, *p* = 0.019), suggesting its potential use in selecting genotypes with a higher number of developing spikes, which is an important yield component. Similarly, the marker Xgwm 285 3B (210bp and 400 bp) showed consistent and significant associations with both spike length (R^2^ = 0.38, *p* = 0.003) and number of grains per spike (R^2^ = 0.44, *p* = 0.0012), highlighting its relevance for improving reproductive traits in wheat. The marker Xgwm 285 showed significant association with grain yield (Table 6).

## Discussion

### Phenotypic analysis

The present study provides compelling evidence of the significant impact of high- heat stress on wheat genotypes that are challenging the wheat production all over the world, especially when exposure occurs during critical growth stages such as heading and grain filling^1,2^. Delayed planting, which subjected genotypes to terminal heat stress, led to notable reductions in grain yield (GY), biological yield (BY), and related agronomic traits in wheat. Lamba et al.^3^ evaluated 50 wheat genotypes to identify the heat stress tolerant genotypes when grown under normal and late sown conditions. The current study revealed notable variability in heat tolerance among the 20 advanced wheat genotypes. Grain yield and kernel weight were the most affected traits under heat stress, showing significant reductions across genotypes. High temperatures are known to shorten the grain-filling duration, resulting in smaller kernels and lower grain weight, which directly contributes to yield loss^3,15–17^. Genotypes such as YR × KSU110-240 exhibited superior performance under stress, maintaining higher grain yield, favorable spike traits, and earlier heading dates. These genotypes demonstrated effective heat tolerance by sustaining yield and stabilizing key agronomic traits under stress. As such, those genotypes hold strong potential for use in breeding programs focused on improving heat-stress resilience in wheat. These results aligned with previous studies suggest that both genetic background and environmental factors critically influence yield and its components under heat stress conditions^1,5,6,15^. High air temperatures during reproductive phases disrupt normal physiological processes, resulting in lower grain size, kernel weight, and thus reduce the grain yield of wheat and other crops^5,14–16^. To cope with that reduction in productivity wheat breeding programs need to prioritize development of heat-tolerant varieties^3^. The results of the current study clearly demonstrated the substantial adverse impact of high-temperature stress on wheat yield and its components under desert environments. Exposure to elevated temperatures, particularly during sensitive stages such as heading and grain filling, significantly reduced both biological and grain yields across genotypes. On average, grain yield decreased by 40.8% (from 6217.9 to 3681.3 kg ha^−1^) and biological yield by 31.2% (from 17,099.2 kg ha^−1^ to 11,760.7 kg ha^−1^) under heat stress, underscoring the vulnerability of wheat production to climate-induced thermal stress. The magnitude of the reduction in biomass is known to depend on the duration and intensity of the stress and when the stress is imposed ^1,4,17^. The results of this study demonstrate that high temperature stress significantly influences key phenological and yield-related traits in wheat, even among genotypes with known heat tolerance. Notably, the heat-tolerant genotype Klassic × KSU105-213 exhibited a reduction in days to heading from 77 days under non-stress conditions to 73 days under high heat stress. This early heading behavior is considered an adaptive trait that allows genotypes to escape terminal heat stress by completing flowering and grain filling before peak temperatures occur. Similar findings have been reported in previous studies, where early flowering was associated with increased heat escape and improved grain yield stability^5,18^. Despite this phenological adjustment, yield components such as the number of grains per spike and 1000-grain weight were negatively impacted by heat stress. In Klassic × KSU105-213, the number of grains per spike declined by approximately 30% under stress, and 1000-grain weight was reduced by 21.6%, indicating that reproductive development and assimilate accumulation were compromised. These reductions are consistent with the physiological effects of heat stress, which can impair pollen viability, reduce photosynthesis, and shorten the grain-filling period^1,19^.

Overall, these findings highlight the complex nature of heat stress responses in wheat and emphasize the importance of multi-trait selection. Genotypes like Klassic × KSU105-213, which combine early flowering with moderately stable yield components under stress, offer promising avenues for breeding programs aimed at improving wheat resilience to rising temperatures.

### Marker-trait associations for heat tolerance in wheat genotypes

The present study explored the genetic diversity and identified marker-trait associations related to terminal heat tolerance in wheat, using a panel of 41 SSR markers distributed across multiple chromosomes^9^ (Gupta et al. 2008). Out of these, 30 markers exhibited polymorphism, confirming their suitability for genetic diversity assessment and QTL validation in wheat. The high level of polymorphism observed among the genotypes demonstrates substantial genetic variability, which is a prerequisite for effective selection in breeding programs. This diversity, as revealed by the dendrogram and Jaccard similarity coefficients (ranging from 0.18 to 1.00), emphasizes the presence of both closely related and genetically distinct genotypes in the tested panel. Such variation is essential for introducing new alleles related to heat stress tolerance and yield stability^9^. Markers with high PIC values are particularly useful for distinguishing genetic variation, and thus be useful candidates for marker-assisted selection (MAS)^7,17^. The clustering of genotypes into three major groups suggests shared genetic backgrounds among many genotypes, especially those grouped in the largest cluster. However, the separation of certain genotypes into distinct clusters (e.g., KSU-110, KSU-115, and Lang × KSU-105-87) indicates potential novel allelic combinations, which could be strategically used in crossing schemes to maximize transgressive segregation for stress-related traits. To identify molecular markers associated with heat stress tolerance, single marker analysis revealed significant associations between several SSR loci and key agronomic traits. The marker Xbarc128-1B was significantly associated with both 1000-kernel weight and days to heading, suggesting its role in influencing grain filling duration and maturity. This dual association enhances its utility for selecting early maturing and productive genotypes, which is vital for escaping terminal heat stress^7,17^. Of particular importance is marker Xgwm577-6B (160 bp), which showed strong associations with multiple traits including 1000-kernel weight, plant height, and biological yield. The high R^2^ values (0.41–0.71) indicate its potential pleiotropic effects or linkage with multiple QTLs governing these traits. Such multi-trait associations make this marker a strong candidate for marker-assisted selection (MAS) to improve yield potential under heat-stressed conditions^13,21^. In addition, the significant correlation of Xgwm 285**-**3B alleles (210 bp and 400 bp) with spike length and number of grains per spike further supports the role of this genomic region in enhancing reproductive capacity under stress. Since these traits are directly tied to sink strength and final yield, markers linked to them can significantly improve selection efficiency for heat-resilient cultivars. Moreover, the validation of 13 SSR alleles as linked to heat tolerance confirms earlier QTL reports and supports their potential application in breeding pipelines. The highest polymorphism information content (PIC) values were recorded for Xgwm577 and Xgwm 285**-** (PIC = 0.38), reinforcing their discriminatory power and genetic informativeness^18,22^. Together, these results provide a strong molecular basis for the selection of genotypes with superior heat tolerance. The identified markers can serve as reliable tools in MAS to enhance the development of heat-resilient wheat varieties by facilitating early selection of key agronomic traits under terminal heat stress. Markers such as Xgwm 285 and Xgwm 577 were identified for their significant association with multiple traits. Xgwm 285, with a PIC of 0.35, an expected heterozygosity (He) of 0.46 and a marker index (MI) of 1.48, proved to be a highly informative marker for identifying genotypes. As well, this marker was associated with spike length and grains per spike, two traits that contributed to reproductive success and overall yield in heat-stressed environments^18^. In current study, Xgwm 577 was linked to important traits such as plant height, 1000 kernels weight, and biological yield, making it a key marker for selecting for high-yielding, early heading date and stable genotypes under high heat stress conditions. Genotypes with early heading date, such as YR × Ksu110-240, showed better yield stability under heat stress. The ability to maintain plant height under heat stress can be crucial for maintaining photosynthetic activity and assimilate distribution during grain filling, thereby stabilizing yields in stressful environments^7,19–21^ The significant role of markers such as Xgwm 285 in regulating both spike length and grains per spike further emphasizes their utility in breeding for improved reproductive traits under heat stress. The association of these markers with reproductive success underscores the importance of selecting genotypes that can maintain spike characteristics, even under unfavorable growing conditions. The genetic proximity of these genotypes suggests that they share beneficial alleles for heat tolerance, making them strong candidates for parent lines in breeding programs aimed at developing heat-tolerant wheat varieties^18^. This genetic diversity, as reflected in the dendrogram, is critical for future breeding programs. By incorporating genetically distinct genotypes into crossing schemes, breeders can maximize the introduction of new alleles that may enhance stress tolerance, yield stability, and adaptability to hot and dry weathers^6,12^.

## Conclusions

High temperature is one of the abiotic stresses that is significantly challenging agricultural production. Heat stress is one of the most critical abiotic factors that is limiting wheat productivity, especially in semi-air and arid regions. The current study evaluated the performance of wheat genotypes under normal and heat-stressed conditions to assess the impact of heat stress on key agronomic traits and to identify heat-tolerant genotypes and associated molecular markers. Significant reductions in grain yield and yield components traits were observed under high heat stress growing conditions, where grain yield decreased by an average of 40.8%. Path analysis indicated negative effect heading date on grain yield under stress conditions. Stress indices indicated that genotype, YR × Ksu110-240, demonstrated a strong resilience under high heat stress, showing only a 31.4% reduction in grain yield, while DHH3-26 showed high sensitivity with a 470.6% grain yield reduction. Simple Sequence Repeat (SSR) markers were used to assess the genetic diversity of these genotypes showed significant marker-trait associations. Markers Xgwm 285 and Xgwm 577 are strongly associated with heat stress tolerance. Those markers and identified genotypes are valuable tools for wheat marker-assisted selection (MAS) programs amid at enhancing wheat resilience under rising global temperatures. The result of this study is providing critical insights for breeding programs aiming to develop wheat varieties that are capable of withstanding heat stress, thereby ensuring stable productivity in the face of climate uncertainty.

## Materials and methods

Twenty different wheat genotypes were used in this study. The advanced breeding genotypes and checks were chosen from heat stress wheat breeding program at plant production department, King Suad university (Supplementary Table 1).

### Morphological, yield and yield components estimation

The 20 wheat genotypes were planted in field trials for two years (2018/2019 and 2019/2020) at the Agricultural Research Station of King Saud University (24° 420 N, 44° 460 E, 400 m asl). For each year, genotypes were planted at two planting dates to control the exposure of wheat plants to high heat stress at heading and grain filling stages. The optimum planting dates (non-stress conditions) were November 15th and 20th in 2019 and 2020, respectively. The Late sowing (high heat stress conditions) were December 15th and 20th in 2020 and 2021, respectively. The late planting date experienced an average temperature ranging from 30.4–31.0/14.2–14.4 °C during the heading and grain-filling stages in both seasons (Supplementary Table 2). The experiment was conducted in split split plot design with three replications, where year was the main plot, the planting date was the sub plot and wheat genotype was the sub sub plot. The plot consisted of five rows, with each spanning 3.0 m and separated by 0.17 m. The seeding rate was 360 seed per square meter, and the fertilizer rates included 180 kg ha^−1^of Nitrogen and 31 kg ha^−1^ of Phosphorus Pentoxide.

Data were collected for morphological and agronomic traits including spike length (SL) in cm, number of kernel per spike (GS), kernel weight (KW 1000) in grams, plant height (PH) in cm, days to heading (DHE), number of spike in ¼ m^2^ (NS), biological yield (BY) and grain yield weight (GY) in kg.ha^−1^. Five plants per plot were randomly harvested from a guarded row for SL, GS), KW, PH, DHE and NS) traits from the central rows, where three average of three samples was calculated to ensure accuracy of calculation. The middle two rows were harvested to assess yield and yield component traits. The differences among wheat genotypes were evaluated for statistical significance using one-way analysis of variance (ANOVA) conducted in STATISTICA^22^ and SAS 9.3 (SAS Institute Inc., Cary, NC) software^23^. Correlation Coefficients using PROC CORR of SAS were used to assess the relationship among measured traits. Path analysis using PROC CALIS of SAS was used to partition the correlation coefficients for measured traits into direct and indirect effects contributing to grain yield. The genotypes were classified according to their heat tolerance level by utilizing the Ward method^24^ and hierarchical cluster analysis. Principal component analysis (PCA) was conducted using JMP 15.2.0 software^29^ to create a scatter plot and factor analysis.

Several equations were used to calculate the tolerance indexes for grain and biological yields including:

Stress tolerance (TOL) = y_C_ − y_HT_^25^,

Stress tolerance index (STI) = (y_C_*y_HT_)/Y_C_^2^^26^,

Yield index (YI) = y_HT_/Y_HT_^27^,

Yield stability index (YSI) = y_HT_/y_C_^28^,

Relative stress index (RSI) = (y_C_/y_HT_)/(Y_C_/Y_HT_)^29^,

Yield Reduction percentage (YRP) = (y_C_ − y_HT_)/y_C_*100^30^,

Stress tolerance efficiency = (y_HT_/y_C_)*100^29^,

Geometric mean productivity (GMP) = √ (y_HT_*y_C_)^26^, and.

Harmonic mean (HM) = 2(y_C_*y_HT_)/(y_C_ + y_HT_ )^31^.

where y_C_ and y_HT_ are the yield of a variety under non-stress(C) and high heat stress (HT), respectively, while Y_C_ and Y_HT_ are the yield mean under non-stress and heat stress conditions, respectively.

### Molecular marker analysis

Genomic DNA was extracted from the seedlings of the 20 wheat genotypes using the modified CTAB method by JJ Doyle and JL Doyle^32^, as outlined by Bellundagi et al.^18^. Leaf samples were harvested and ground in liquid nitrogen. The DNA quality was assessed by 0.8% agarose gel electrophoresis, and quantification was carried out using a nanodrop.

Thirty SSR markers were selected based on their association with QTL markers for heat stress in wheat^33–35^ (Supplementary tables 3 and 4). PCR reaction was performed in PCR reactions, visualization and scoring were conducted according to Ghazy et al.^36^. SSR amplification profiles were scored visually, as homozygous tolerant, homozygous sensitive, and heterozygous. The PowerMarker software^37^ was utilized to calculate the number of alleles and the level of polymorphic information content (PIC)^38^.

Single marker analysis was carried out by fitting the SSR data (as independent variable) and the phenotypic data (as dependent variable) using single linear regression model of SAS software^23^. The percentage of variation of each of phenotypic trait were also estimated.

## Supplementary Information

Below is the link to the electronic supplementary material.

Supplementary Material 1

## Data Availability

The datasets used and/or analyzed during the current study available from the corresponding author on reasonable request.
