# Supplementary material for: Selection of heat stress tolerant wheat genotypes for desert environments
Source: Sci Rep. 2025 Oct 21;15:36672. doi: 10.1038/s41598-025-20450-7 (PMC12541005; doi:10.1038/s41598-025-20450-7)
Supplement: Supplementary file 1 — Supplementary Material 1 [file 41598_2025_20450_MOESM1_ESM.docx]

| **Supplementary Table 1. Description of the wheat genotypes used in this study.** | | |  |
| --- | --- | --- | --- |
|  | Genotype | Pedigree | Source |
| 1 | Lang x Ksu105-87 | QT3765/Sunco x HD 2172\ RI 474 - 87 | KSU |
| 2 | Lang x Ksu105-47 | QT3765/Sunco x HD 2172\ RI 474 - 47 | KSU |
| 3 | YR x Ksu110-190 | CIANO-67//SONORA-64/KLEIN-RENDIDOR/3/II-8156 x Sama\ Yecora Rojo-L11-6 -190 | KSU |
| 4 | YR x Ksu110-240 | CIANO-67//SONORA-64/KLEIN-RENDIDOR/3/II-8156 x Sama\ Yecora Rojo-L11-6 -240 | KSU |
| 5 | YR x Ksu110-277 | CIANO-67//SONORA-64/KLEIN-RENDIDOR/3/II-8156 x Sama\ Yecora Rojo-L11-6 -277 | KSU |
| 6 | YR x Lang-4 | CIANO-67//SONORA-64/KLEIN-RENDIDOR/3/II-8156 x QT3765/Sunco - 4 | KSU |
| 7 | YR x Lang-15 | CIANO-67//SONORA-64/KLEIN-RENDIDOR/3/II-8156 x QT3765/Sunco - 15 | KSU |
| 8 | YR x Lang-30 | CIANO-67//SONORA-64/KLEIN-RENDIDOR/3/II-8156 x QT3765/Sunco - 30 | KSU |
| 9 | YR x Lang-60 | CIANO-67//SONORA-64/KLEIN-RENDIDOR/3/II-8156 x QT3765/Sunco - 60 | KSU |
| 10 | YR x Lang-66 | CIANO-67//SONORA-64/KLEIN-RENDIDOR/3/II-8156 x QT3765/Sunco - 66 | KSU |
| 11 | Klasic x Ksu105-11 | KLEIN-RENDIDOR/2*SONORA-64//INIA-66/3/CIANO-67/4/YECORA-70 x HD 2172\ RI 474 - 11 | KSU |
| 12 | Klasic x Ksu105-213 | KLEIN-RENDIDOR/2*SONORA-64//INIA-66/3/CIANO-67/4/YECORA-70 x HD 2172\ RI 474 - 13 | KSU |
| 13 | Lang | QT3765/Sunco | AUSTRALIA |
| 14 | Yecora Rojo | CIANO-67//SONORA-64/KLEIN-RENDIDOR/3/II-8156 | USA |
| 15 | Klasic | KLEIN-RENDIDOR/2*SONORA-64//INIA-66/3/CIANO-67/4/YECORA-70 | USA |
| 16 | Ksu105 | HD 2172\ RI 474 | KSU |
| 17 | Ksu110 | Sama\ Yecora Rojo-L11-6 | KSU |
| 18 | Ksu115 | Sama\ Yecora Rojo-L11-21 | KSU |
| 19 | DHH3-26 | Klasic x Ksu105 - 26 | KSU |
| 20 | DHH3-76 | Klasic x Ksu105 - 76 | KSU |

| **Supplementary Table 2: Average monthly temperatures, humidity, and precipitation** | | | | | |
| --- | --- | --- | --- | --- | --- |
| Weather Data for Riyadh (2018/2019 | |  |  |  |  |
| **Month** | **Relative Humidity (%)** | **Precipitation (mm)** | **Average Temperature (°C)** | **Minimum Temperature** | **Maximum Temperature** |
|  |  |  |  | **(°C)** | **(°C)** |
| November | 30-35 | 10-May | 21.5 | 15 | 22 |
| December | 40-45 | 10-May | 16.5 | 11 | 22 |
| January | 45- 50 | 10-May | 14.5 | 9 | 20 |
| February | 35-45 | 10-May | 18 | 12 | 24 |
| March | 30-35 | 10-May | 22.5 | 16 | 29 |
| April | 15-25 | 5-Jan | 28.5 | 22 | 35 |
| May | 10-15 | 0-3 | 33.5 | 27 | 40 |
|  |  |  |  |  |  |
| Weather Data for Riyadh (2019/2020) | |  |  |  |  |
| **Month** | **Relative Humidity (%)** | **Precipitation (mm)** | **Average Temperature (°C)** | **Minimum Temperature** | **Maximum Temperature** |
|  |  |  |  | **(°C)** | **(°C)** |
| November | 30-35 | 10-May | 21.5 | 14 | 29 |
| December | 40-45 | 10-May | 15.5 | 10 | 21 |
| January | 45-50 | 10-May | 13.5 | 8 | 19 |
| February | 35-40 | 10-May | 17 | 11 | 23 |
| March | 30-35 | 10-May | 21.5 | 15 | 28 |
| April | 15-25 | 5-Jan | 28.5 | 21 | 36 |
| May | 10-15 | 0-3 | 33.5 | 26 | 41 |

Sources: Data sources: Meteoblue , Time and Date and <https://www.weather-atlas.com/en/saudi-arabia/riyadh-climate#google_vignette>

**Supplementary Table 3**: Characterization of microsatellite markers localized on chromosomes

| Ta (°C) | Reverse Primer | Forward Primer | Position | Maker | Sr. No |
| --- | --- | --- | --- | --- | --- |
| 57 | GCCCCAACCACCTCCC | GGCACCGATGCTTCCA | 2B | Xbarc10 | 1 |
| 57 | CACATGGCATCACATTTGTG | AAACCATATTGGGAGGAAAGG | 2A | Xgwm448 | 2 |
| 57 | CAAACCAGGCAAGAGTCTGA | GCGGGTAGCATTTATGTTGA | 1B | Xbarc128 | 3 |
| 57 | GGCCCATTTCCCACTTTCCA | CCAGCCCCTCTACACATTTT | 1B | Xbarc137 | 4 |
| 57 | GGCCTGTCAATTATGAGC | CCGGTGAGAGGACTAAAA | 5A,6A,2D | Xbarc142 | 5 |
| 55 | TACCCTGATGCTGTAATATGTG | GTGAGCAATTTTGATTATACTG | 1A | WMC24 | 6 |
| 57 | ACGTGCTAGGGAGGTATCTTGC | GAGGGTTCTGAAATGTTTTGCC | 4B | Xwmc48 | 7 |
| 57 | CGCCTCTAGCGAGAGCTATG | GCAGAGCCTGGTTCAAAAAG | 5A | Xgwm186 | 8 |
| 57 | CCC AAT TTT GCT AAG TTC ACT | GCG TAT GTA TGT CTA TTT TCC TAT CT | 3A | Xbarc1044 | 9 |
| 55 | CAGTGCCAAAATGTCGAAAGTC | ATCACAATGCTGGCCCTAAAAC | 7B | Xwmc364 | 10 |
| 57 | GTGAATTGTGTCTTGTATGCTTCC | GGATAGTCAGACAATTCTTGTG | 1B | Xgwm11 | 11 |
| 57 | AATGCAAAGTGAAAAACCCG | GATCAAACACACACCCCTCC | 2A | Xgwm95 | 12 |
| 57 | AGTCGCCGTTGTATAGTGCC | CGACCCGGTTCACTTCAG | 5A | Xgwm205 | 13 |
| 57 | GGTAACCACTAGAGTATGTCCTT | AGTTATGTATTCTCTCGAGCCTG | 7A | Xwmc273 | 14 |
| 55 | ATCGACCGGGATCTAGCC | ATGACCCTTCTGCCAAACAC | 3B | Xgwm285 | 15 |
| 55 | CAAAGCTTGACTCAGACCAAA | GTGAGGCAGCAAGAGAGAAA | 2B | Xgwm148 | 16 |
| 55 | GTGAATTGTGTCTTGTATGCTTCC | GGATAGTCAGACAATTCTTGTG | 7B | Xgwm11 | 17 |
| 57 | TCGCCATCACTCGTTCAAG | TACTGGTTCACATTGGTGCG | 5A | Xgwm293 | 18 |
| 55 | GCCATCCGTTGACTTGAGGTTA | CTCATCGTCCTCCTCCACTTTG | 2A | Xwmc658 | 19 |
| 55 | TGCTAACTGGCCTTTGCC | CCTCTTCCTCCCTCACTTAGC | 1B | Xgwm337 | 20 |
| 58 | GTGCCACGTGGTACCTTTG | GTGCTTGCTGAGCTATGAGTC | 1B | Xgwm190 | 21 |
| 57 | CGGTCCAAGTGCTACCTTTC | CGTACTCCACTCCACACGG | 5B | Xgwm335 | 22 |
| 55 | TGTTTCAAGCCCAACTTCTATT | ATGGCATAATTTGGTGAAATTG | 7B | Xgwm577 | 23 |
| 55 | TCTGTAGGCTCTCTCCGACTG | ACCTGATCAGATCCCCATCG | 7B | Xgwm611 | 24 |
| 55 | CTC CGA TGG ATT ACT CGC AC | GAT CTT GGC GCT GAG AGA GA | 6A | Xgwm617 | 25 |
| 57 | AGCGTTCTTGGG AATTAGAGA | CCAATCAGCCTG CAACAAC | 7A,7D | Xgwm635 | 26 |
| 57 | TGC TGC TGG TCT CTG TG | GCA CCC ACA TCT TCG ACC | 3A | Xgwm666 | 27 |
| 55 | GCCATCCGTTGACTTGAGGTTA | CTCATCGTCCTCCTCCACTTTG | 7AL | Xwmc65 | 28 |
| 57 | TGTGTGTGTGTGTGTCTCTCTC | TCAGGGACACAAATAAATTGATACC | 2A | Xgwm205 | 29 |
| 57 | TAATGGTGGATCCATGATAGCC | ACGTATCCAGACACTGTGGTAA | 1DL | Xwmc216 | 30 |

**Supplementary Table 4: Summary of the characterization of SSR molecular markers for H: expected heterozygosity; PIC: polymorphism information content; E: effective multiplex ratio; H_av: mean heterozygosity; MI: marker index; and D: discriminating power.**

| Primer Name | Chr. No. | # Amplified bands. | # Polymorphic (PM) | # alleles | PM % | H | PIC | E | H.av | MI | D |
| --- | --- | --- | --- | --- | --- | --- | --- | --- | --- | --- | --- |
| Xbarc 10 | 2B | 1 | 1 | 17 | 100 | 0.26 | 0.22 | 0.85 | 0.0128 | 0.0108 | 0.28 |
| Xbarc 128 | 1B | 3 | 2 | 40 | 66.67 | 0.44 | 0.35 | 2.00 | 0.0074 | 0.0148 | 0.56 |
| Xbarc 137 | 1B | 1 | 1 | 12 | 100 | 0.48 | 0.36 | 0.60 | 0.0240 | 0.0144 | 0.65 |
| Xbarc 142 | 5A | 1 | 1 | 7 | 100 | 0.46 | 0.35 | 0.35 | 0.0228 | 0.0080 | 0.89 |
| Xbarc 1044 | 3A | 1 | 1 | 19 | 100 | 0.10 | 0.09 | 0.95 | 0.0048 | 0.0045 | 0.10 |
| Xgwm 11 | 1B | 4 | 3 | 49 | 75 | 0.47 | 0.36 | 2.45 | 0.0059 | 0.0145 | 0.63 |
| Xgwm 205 | 5A | 1 | 0 | 20 | 0 | 0.00 | 0.00 | 1.00 | 0.0000 | 0.0000 | 0.00 |
| Xgwm 285 | 3B | 2 | 1 | 26 | 50 | 0.46 | 0.35 | 1.30 | 0.0114 | 0.0148 | 0.58 |
| Xgwm 337 | 1D | 1 | 1 | 14 | 100 | 0.42 | 0.33 | 0.70 | 0.0210 | 0.0147 | 0.52 |
| Xgwm 577 | 6B | 2 | 2 | 20 | 100 | 0.50 | 0.38 | 1.00 | 0.0125 | 0.0125 | 0.76 |
| Xgwm 611 | 7B | 2 | 2 | 10 | 100 | 0.38 | 0.30 | 0.50 | 0.0094 | 0.0047 | 0.94 |
| Xgwm 617 | 7D | 3 | 3 | 25 | 100 | 0.49 | 0.37 | 1.25 | 0.0081 | 0.0101 | 0.83 |
| Xgwm 635 | 7A | 2 | 2 | 27 | 100 | 0.44 | 0.34 | 1.35 | 0.0110 | 0.0148 | 0.55 |
| Xgwm 666 | 3A | 2 | 2 | 28 | 100 | 0.42 | 0.33 | 1.40 | 0.0105 | 0.0147 | 0.52 |
| Xgwm 65 | 7A | 1 | 1 | 18 | 100 | 0.18 | 0.16 | 0.90 | 0.0090 | 0.0081 | 0.19 |
| Xwmc 170 | 2D | 2 | 2 | 20 | 100 | 0.50 | 0.38 | 1.00 | 0.0125 | 0.0125 | 0.76 |
| Xwmc 216 | 1D | 2 | 2 | 28 | 100 | 0.42 | 0.33 | 1.40 | 0.0105 | 0.0147 | 0.52 |
| Total |  | 31 | 27 | 380 |  |  |  |  |  |  |  |
| AV/Primer |  | 1.82 | 1.59 | 22.35 | 87.75 | 0.38 | 0.29 | 1.12 | 0.0114 | 0.0111 | 0.55 |

| **Supplementary Table 5: Markers classes (0,1) means and standard deviation for significant SSR markers associate with SL: spike length; GS number of kernel per spike; GW: 1000 kernel weight (g); PH: plant height(cm); DHE: number of days to heading; NS: number of spike per ¼ square meter and BY: biological yield and GY: grain yield (Kg.Ha-1) for what genotypes growing under heat stress conditions (S)** | | | | | | | | | | | | | | | |
| --- | --- | --- | --- | --- | --- | --- | --- | --- | --- | --- | --- | --- | --- | --- | --- |
|  |  |  |  |  |  |  |  |  |  |  |  |  |  |  |  |
| **Level of** | **GW_S** | |  | **DHE_S** | |  |  |  |  |  |  |  |  |  |  |
| **Xbarc 128-1B - 210bp** | **Mean** | ***SD*** |  | **Mean** | ***SD*** |  |  |  |  |  |  |  |  |  |  |
| **0** | 37.78 | *2.86* |  | 67.87 | *3.81* |  |  |  |  |  |  |  |  |  |  |
| **1** | 33.70 | *3.76* |  | 73.88 | *6.68* |  |  |  |  |  |  |  |  |  |  |
|  |  |  |  |  |  |  |  |  |  |  |  |  |  |  |  |
| **Level of** | **GW_S** | |  |  |  |  |  |  |  |  |  |  |  |  |  |
| **Xbarc 128-1B - 240bp** | **Mean** | ***SD*** |  |  |  |  |  |  |  |  |  |  |  |  |  |
| **0** | 34.23 | *3.61* |  |  |  |  |  |  |  |  |  |  |  |  |  |
| **1** | 37.84 | *2.95* |  |  |  |  |  |  |  |  |  |  |  |  |  |
|  |  |  |  |  |  |  |  |  |  |  |  |  |  |  |  |
| **Level of** | **SL_S** | |  |  |  |  |  |  |  |  |  |  |  |  |  |
| **Xgwm 11-1B - 280bp** | **Mean** | ***SD*** |  |  |  |  |  |  |  |  |  |  |  |  |  |
| **0** | 9.73 | *1.07* |  |  |  |  |  |  |  |  |  |  |  |  |  |
| **1** | 10.40 | *1.99* |  |  |  |  |  |  |  |  |  |  |  |  |  |
|  |  |  |  |  |  |  |  |  |  |  |  |  |  |  |  |
| **Level of** | **SL_S** | |  | **GS_S** | |  | **NS_S** | |  | **GY_S** | |  |  |  |  |
| **Xgwm 285-3B - 400bp** | **Mean** | ***SD*** |  | **Mean** | ***SD*** |  | **Mean** | ***SD*** |  | **Mean** | ***SD*** |  |  |  |  |
| **0** | 10.60 | *1.65* |  | 46.81 | *7.32* |  | 32.34 | *5.95* |  | 3545.33 | *839.95* |  |  |  |  |
| **1** | 9.09 | *0.51* |  | 41.25 | *1.17* |  | 34.90 | *4.65* |  | 3886.38 | *536.51* |  |  |  |  |
|  |  |  |  |  |  |  |  |  |  |  |  |  |  |  |  |
| **Level of** | **SL_S** | |  | **GW_S** | |  | **PH_S** | |  | **BY_S** | |  | **GY_S** | |  |
| **Xgwm 577-6B - 180bp** | **Mean** | ***SD*** |  | **Mean** | ***SD*** |  | **Mean** | ***SD*** |  | **Mean** | ***SD*** |  | **Mean** | ***SD*** |  |
| **0** | 11.10 | *2.20* |  | 40.20 | *2.05* |  | 73.48 | *4.03* |  | 9438.00 | *458.22* |  | 3046.00 | *462.18* |  |
| **1** | 9.63 | *1.04* |  | 35.61 | *3.13* |  | 89.39 | *4.78* |  | 12535.00 | *1010.16* |  | 3893.67 | *696.15* |  |
|  |  |  |  |  |  |  |  |  |  |  |  |  |  |  |  |
| **Level of** | **SL_S** | |  | **GW_S** | |  | **PH_S** | |  | **BY_S** | |  | **GY_S** | |  |
| **Xgwm 577-6B - 160bp** | **Mean** | ***SD*** |  | **Mean** | ***SD*** |  | **Mean** | ***SD*** |  | **Mean** | ***SD*** |  | **Mean** | ***SD*** |  |
| **0** | 9.63 | *1.04* |  | 35.61 | *3.13* |  | 89.39 | *4.78* |  | 12535.00 | *1010.16* |  | 3893.67 | *696.15* |  |
| **1** | 11.10 | *2.20* |  | 40.20 | *2.05* |  | 73.48 | *4.03* |  | 9438.00 | *458.22* |  | 3046.00 | *462.18* |  |
|  |  |  |  |  |  |  |  |  |  |  |  |  |  |  |  |
| **Level of** | **NS_S** | |  |  |  |  |  |  |  |  |  |  |  |  |  |
| **Xgwm 611-7B - 170bp** | **Mean** | ***SD*** |  |  |  |  |  |  |  |  |  |  |  |  |  |
| **0** | 32.14 | *4.99* |  |  |  |  |  |  |  |  |  |  |  |  |  |
| **1** | 40.33 | *0.96* |  |  |  |  |  |  |  |  |  |  |  |  |  |
|  |  |  |  |  |  |  |  |  |  |  |  |  |  |  |  |
| **Level of** | **SL_S** | |  |  |  |  |  |  |  |  |  |  |  |  |  |
| **Xgwm 611-7B - 190bp** | **Mean** | ***SD*** |  |  |  |  |  |  |  |  |  |  |  |  |  |
| **0** | 10.47 | *1.64* |  |  |  |  |  |  |  |  |  |  |  |  |  |
| **1** | 9.11 | *0.57* |  |  |  |  |  |  |  |  |  |  |  |  |  |
|  |  |  |  |  |  |  |  |  |  |  |  |  |  |  |  |
| **Level of** | **GS_S** | |  |  |  |  |  |  |  |  |  |  |  |  |  |
| **Xgwm 617-7D - 130bp** | **Mean** | ***SD*** |  |  |  |  |  |  |  |  |  |  |  |  |  |
| **0** | 47.26 | *6.64* |  |  |  |  |  |  |  |  |  |  |  |  |  |
| **1** | 40.58 | *2.60* |  |  |  |  |  |  |  |  |  |  |  |  |  |
